# Generation of realistic virtual adult populations using a model-based copula approach

**DOI:** 10.1007/s10928-024-09929-4

**Published:** 2024-06-06

**Authors:** Yuchen Guo, Tingjie Guo, Catherijne A. J. Knibbe, Laura B. Zwep, J. G. Coen van Hasselt

**Affiliations:** 1 Systems Pharmacology and Pharmacy, Leiden Academic Center for Drug Research, Leiden University, Einsteinweg 55, Leiden, 2333 CC The Netherlands; 2 Department of Clinical Pharmacy, St. Antonius Hospital, Nieuwegein, The Netherlands

**Keywords:** Copula, Virtual population, Covariate modeling, Pharmacometric simulation

## Abstract

Incorporating realistic sets of patient-associated covariates, i.e., virtual populations, in pharmacometric simulation workflows is essential to obtain realistic model predictions. Current covariate simulation strategies often omit or simplify dependency structures between covariates. Copula models are multivariate distribution functions suitable to capture dependency structures between covariates with improved performance compared to standard approaches. We aimed to develop and evaluate a copula model for generation of adult virtual populations for 12 patient-associated covariates commonly used in pharmacometric simulations, using the publicly available NHANES database, including sex, race-ethnicity, body weight, albumin, and several biochemical variables related to organ function. A multivariate (vine) copula was constructed from bivariate relationships in a stepwise fashion. Covariate distributions were well captured for the overall and subgroup populations. Based on the developed copula model, a web application was developed. The developed copula model and associated web application can be used to generate realistic adult virtual populations, ultimately to support model-based clinical trial design or dose optimization strategies.

**Supplementary Information:**

The online version contains supplementary material available at 10.1007/s10928-024-09929-4.

## Introduction

In pharmacometric modeling, patients’ covariates are usually identified as a source of variability between individuals that impacts pharmacokinetics and pharmacodynamics [1]. Generation of virtual populations (VPs), i.e., realistic sets of patient characteristics or covariates, is essential to ensure that realistic responses are produced in pharmacometric simulations, eventually providing valuable information to support *in silico* clinical trials and optimization of dosing strategies.

Realistic VPs should reflect both the marginal distribution and dependency structure observed between covariate variables of interest. In statistics, a marginal distribution describes the probability distribution of one separate variable, and a dependence structure reveals the relationships or dependent patterns between variables in a dataset. For instance, within a real-world dataset focusing on the elderly population, age, as a covariate, may exhibit a t-distributed margin, with a certain mean and standard deviation; meanwhile, it could be negatively correlated with renal function biomarkers. Misspecification of the margins or dependency structures, i.e., in comparison with those actually observed, may impact the quality of subsequent patient responses obtained in pharmacometric simulations.

VPs can be generated using several approaches, which are either data-driven or distribution-driven. Data-driven methodologies such as the bootstrap or conditional distribution modeling [2] utilize an actual dataset of patient characteristics to sample from. Requesting such data, however, is sometimes not possible due to patient privacy regulations. Distribution-based approaches characterize the distribution of the marginals of covariates of interest but may not always capture their dependency structure. For example, series of univariate distributions can be used to describe the marginals yet ignore interdependencies between covariates. Multivariate normal distributions [3] do consider the dependency but assume that variables are normally distributed, which may not always hold. Finally, machine learning algorithms [4–6] have been proposed, but these models are usually based on complex frameworks and often lack interpretability of underlying dependencies.

Copula modeling is a powerful tool for calculating multivariate distributions and has been widely used in various fields, such as finance [7–9], climate research [10, 11] and engineering [12, 13]. Copulas can capture the dependence structure between random variables independently from the description of the marginals [14]. Using a transformation of any marginal distribution to a uniform distribution, the dependence structure can be separated from the marginal structure. Moreover, a rich variety of copula models is available to be selected to estimate diverse dependent patterns in data [15]. An extension of the copula, the vine copula, addresses the difficulty of calculating multivariate joint distributions by using conditional dependence and bivariate building blocks [16]. Recently, copulas have been introduced to the field of pharmacometrics as a relevant key strategy for VP generation, demonstrating favorable performance in simulating realistic VPs compared to standard approaches, while their distribution-based nature facilitates sharing of covariate data within the community [17].

Here, we present a copula model for the simulation of adult virtual populations. We first developed a copula model for 12 covariates of relevance for pharmacometric models using data from adult individuals present in the NHANES database [18]. Then we evaluated the performance of the copula in simulating the overall and subgroup populations. Finally, a web application was designed for the copula model developed to facilitate generation of adult VPs.

## Methods

### Data

We used the public database from National Health and Nutrition Examination Survey (NHANES), an initiative that collects data on non-institutionalized individuals in the U.S., including laboratory measurements, physical screening, and surveys; data are released to the public every two years [18]. We combined the NHANES data for 2009 ∼ 2010, 2011 ∼ 2012, 2013 ∼ 2014, 2015 ∼ 2016, and 2017 ∼ 2018 releases based on their accessibility and consistency in laboratory methods. Differences in laboratory, instruments, and methods across releases were considered by implementing the adjustment equations provided by NHANES.

We focused on the adult population aged 18–80 years, with 27,008 subjects in total. Common covariates of interest for population pharmacokinetic models were selected: sex, race-ethnicity, age, height, body weight, fat mass (Fat), serum creatinine (SCR), alanine aminotransferase (ALT), aspartate aminotransferase (AST), alkaline phosphatase (ALP), albumin and total bilirubin (BR) [19–23]. We acknowledge the sensitivity regarding the use of race-ethnicity as a medical indicator. Its inclusion in our study is focused on subgroup analysis when relevant, and not intended to perpetuate stereotypes or contribute to health disparities.

Table 1 provides the summary statistics of covariates in the model development dataset. Of note, over 50% of fat mass data in the observed dataset were missing. Fat mass is measured via dual-energy x-ray absorptiometry (DXA) examination. Half of the missing data were due to not meeting the inclusion criteria of age (< 60 years old) during the DXA examination, while another half were due to the examination not being conducted in the 2009 ∼ 2010 release. Since copulas allow to be estimated from incomplete datasets and generate complete simulation datasets, a validation analysis was conducted (supplementary material) to provide more insights into the reliability of simulated fat mass for people aged  ≥ 60 years. This analysis involved validating fat mass predictions after excluding measured fat mass data for individuals within specific age groups. The result showed no significant bias in the simulated fat mass for the age groups under 60 years old (Figure S1).

**Table 1:** Summary statistics of covariates in dataset combined from National Health and Nutrition Examination Surveys 2009 ∼ 2010, 2011 ∼ 2012, 2013 ∼ 2014, 2015 ∼ 2016 and 2017 ∼ 2018. The total number of individuals was *n* = 27,008

Variable name	Variable description	Percentage (%)	Actual *N* (% Missing)	Mean$\pm$ SD [range]
Sex	*Gender*	**-**	27,008 (0%)	-
	Male	48.5	13,104	-
	Female	51.5	13,904	-
Race- Ethnicity	*Race-Ethnicity*	-	27,008 (0%)	-
	Hispanic*	26.6	7176	-
	White	36.9	9978	-
	African American	22.8	6166	-
	Asian	10.6	2859	-
	Other Race	3.1	829	
Age	Age (year)		27,008 (0%)	46.05 ± 17.21 [18 ∼ 79]
Weight	Body weight (kg)		26,746 (1.0%)	82.02 ± 22.17 [32.3 ∼ 242.6]
Height	Standing height (cm)		26,757 (1.0%)	167.16 ± 10.09 [123.3 ∼ 204.5]
Fat	Total body fat (kg)		11,826 (56.2%)	27.11 ± 11.93 [4.9 ∼ 102.3]
SCR	Serum creatinine (mg/dL)		25,313 (6.3%)	0.88 ± 0.45 [0.16 ∼ 17.41]
ALT	Alanine aminotransferase (ALT, U/L)		25,307 (6.3%)	25.57 ± 20.42 [5 ∼ 1363]
AST	Aspartate aminotransferase (AST, U/L)		25,288 (6.4%)	25.93 ± 17.13 [7 ∼ 882]
ALP	Alkaline phosphatase (ALP, U/L)		25,310 (6.3%)	69.34 ± 24.59 [7 ∼ 907]
Albumin	Albumin (g/dL)		25,315 (6.3%)	4.28 ± 0.35 [2.0 ∼ 5.6]
BR	Total bilirubin (mg/dL)		25,298 (6.3%)	0.62 ± 0.31 [0.0 ∼ 7.3]

* Mexican American and other Hispanic in NHANES were recorded as Hispanic in our real-world dataset. Other race-ethnicity groups remained unchanged

### Vine copula model development

A vine copula was fitted to the NHANES data. First, to avoid producing covariates of negative values in VPs, biochemical measurement data were log-transformed. As copulas are joint distribution functions with uniform margins, data were then transformed into uniform distributions using the probability integral function [24] based on kernel density estimation. Candidate vine copula models consist of parametric bivariate copula functions, such as Gaussian, Clayton and Frank copulas. Each kind of bivariate copula possesses distinct strengths in depicting various dependence behaviors, and a rich variety of bivariate copulas are available to be selected to represent diverse dependence patterns in data.

The vine copula model was constructed with a tree structure, which defines the pairs of covariates and copulas to be estimated. A vine tree structure comprises a sequence of trees, with the first tree representing a group of unconditional bivariate copulas, and subsequent trees representing a group of bivariate copulas conditional on the previous trees. Each edge in a tree represents a bivariate copula between two covariates (the first tree) or two copulas (higher order trees).

The vine tree structure was sequentially selected and estimated. For the first tree, the maximum spanning tree (MST) algorithm was used to select covariate pairs in each tree by maximizing the sum of correlations over the possible pairs in each tree, then all bivariate copula functions (Gaussian, Clayton or other bivariate distribution functions) were fit for the selected pairs and parameters were estimated; the best-fitting bivariate functions were selected based on the Akaike Information Criterion (AIC). This procedure was iterated for each subsequent tree until all trees were selected and estimated. Detailed methodology was described in the literature on bivariate copulas ([25] C3), tree structures ([25] C5), and MST [26].

To incorporate the covariate ‘race-ethnicity’ in the copula and optimize the model, we treated race-ethnicity as an ordered categorical variable and tested copulas with all order combinations.

### Model evaluation

Model evaluation was conducted through a simulation-based strategy: performing 100 simulations of the original dataset and comparing the metrics between the real-world population and VPs that were back transformed to their original scales. To assess the model performance on the marginal distributions, we evaluated observed and simulated populations by comparing the frequency of each category for categorical covariates, and for continuous covariates, comparing the marginal metrics, mean, standard deviation (SD), and percentiles (5th, 50th and 95th ), denoted by *M*, between observed and simulated data in terms of relative error (*RE*) (Eq. 1).

1 $$RE=\frac{{M}_{sim}-{M}_{obs}}{{M}_{obs}}$$

where ${M}_{sim}$ and ${M}_{obs}$ represent the metrics for simulated population and observed population, respectively.

To assess the performance of the model on capturing the dependency structure, pairwise correlation coefficients were compared between observed and simulated datasets. Since data sharing the same correlation could display various shapes of the dependence, a two-dimensional metric was developed to quantify the overlap of the density contours in observed and simulated data. For each pair combination of covariates, 95th percentile density contours were calculated for observed and simulated populations. The overlap metric was computed as the Jaccard index [27]: the ratio between the intersection area and union area (Figure S2). Higher overlap indicated a better description of dependence relations. We systematically evaluated the performance of the model from the following aspects:

1. Overall performance: the NHANES copula (full copula) was developed based on the whole set of participants of NHANES that represents a general population. Simulated populations and the real-world population were then compared.
2. Subgroup performance: populations of interest in clinical trials and cohort studies typically comprise individuals with certain race-ethnicity or sex. To be able to create realistic VP of interest, it is important to determine whether the full copula could capture the characteristics of subgroup populations. Predictive performance of the full copula for subsets of VPs was assessed with a particular interest in the race-ethnicity and sex subgroups. For comparison, two series of subgroup copulas were also constructed using data specific of each subgroup population:1) Hispanic copula, White copula, African American copula, Asian copula, Other race copula, 2) male copula, female copula. Virtual subgroup populations were obtained in two ways: by simulating from the full copula model and filtering out the irrelevant individuals, and by directly simulating from the subgroup copula. The performance of full copula was compared with that of subgroup copula to provide an understanding of whether the full copula was sufficient for generating subsets of VPs.

### Shiny application development

To provide a convenient and user-friendly tool, an interactive web application that could output VPs was developed using the NHANES copula. Next to the NHANES copula, a weighted copula was estimated with the incorporation of sampling weights [28] to address the sampling bias in NHANES. The sample weights account for complex sampling design and non-response of NHANES and are associated with demographic properties of the US population. The weighted copula allows users to sample a virtual population that is representative of the actual US population.

### Software

The analysis was performed in R 4.1.2. Processing of NHANES data was conducted with *survey* package. Kernel density estimation of marginal distributions was performed with *kde1d* package. Development of NHANES copula was implemented with *rvinecopulib* package. The overlap metric was calculated using *ks* and *sf* packages. R shiny application was developed using *shiny* package. Visualizations of this study were generated with *ggplot2* package. All scripts are available on https://github.com/vanhasseltlab/NHANES_copula.

## Results

### Vine copula of NHANES data

**Fig. 1:**
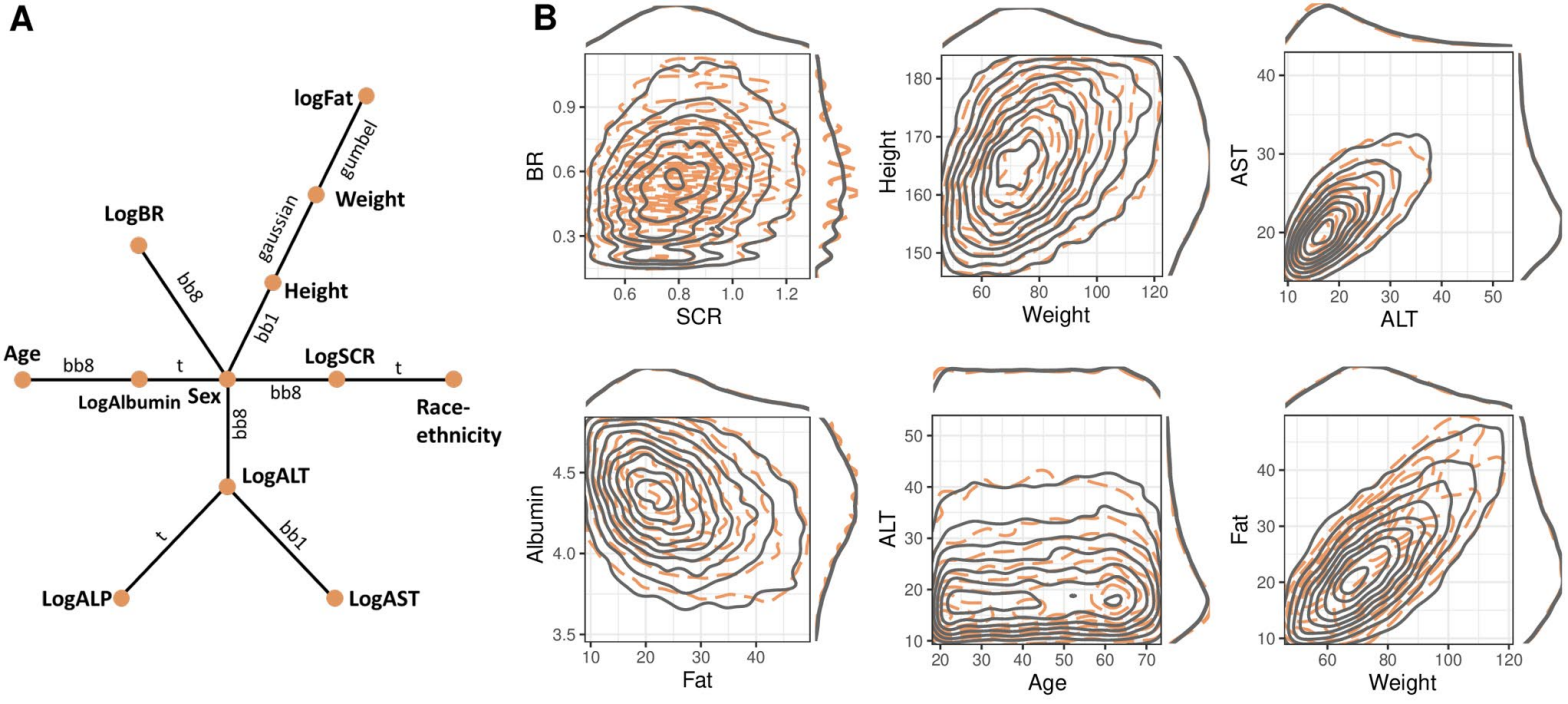
Graphical representation of dependence structure estimated by NHANES copula and bivariate densities of observed and simulated covariates. **A**. The first tree structure of NHANES copula with 12 covariates (nodes), and 11 copulas (edges). The texts on the edges denote the selected bivariate distribution functions: Gaussian, Gumbel, t, Clayton-Gumbel (bb1) and Joe-Frank (bb8). The tree was chosen based on the maximum spanning tree algorithm. The inter-dependent relationships between other covariate pairs were captured by subsequent trees that describe the conditional dependency. **B**. Density contours of different covariate pairs of the observed population (orange dashed line) and the simulated population using the NHANES copula (gray solid lines). Marginal densities were displayed on the top and right sides of each plot. Six covariate pairs were chosen to present the diverse dependent patterns as illustrative examples. Abbreviations: LogFat: log fat mass; LogSCR: log serum creatinine concentration (SCR); LogALT: log alanine aminotransferase concentration (ALT); LogAST: log aspartate aminotransferase concentration (AST); LogALP: log alkaline phosphatase concentration (ALP); LogAlbumin: log albumin concentration; LogBR: log total bilirubin concentration (BR)

Logarithmic and uniform transformed data were fitted to estimate the underlying dependency structure with a vine copula. Instead of displaying the whole tree structure, we only showed the first tree since the first layer dependence captured the strongest correlations while trees of higher levels describe the conditional dependence, and are less influential on the overall fit than the first tree [29]. Sex was located at the center of the first tree structure, as it showed relatively strong dependence relationships with height, logBR, logALT, logAlbumin, and logSCR (Fig. 1A). The density contours of covariate pairs in the real-world population displayed various patterns, and the VP was found to overlap the real-world population in selected covariate pairs well (Fig. 1B).

Estimating the NHANES copula using the input dataset comprising 27,008 records with 12 covariates required approximately 20 min on a Windows computer with Intel Core i7 processor operating at 2.80 GHz. In contrast, simulations from copula are computationally efficient, with an average of 1.6 s per 1000 individuals simulated.

### Overall performance

**Fig. 2:**
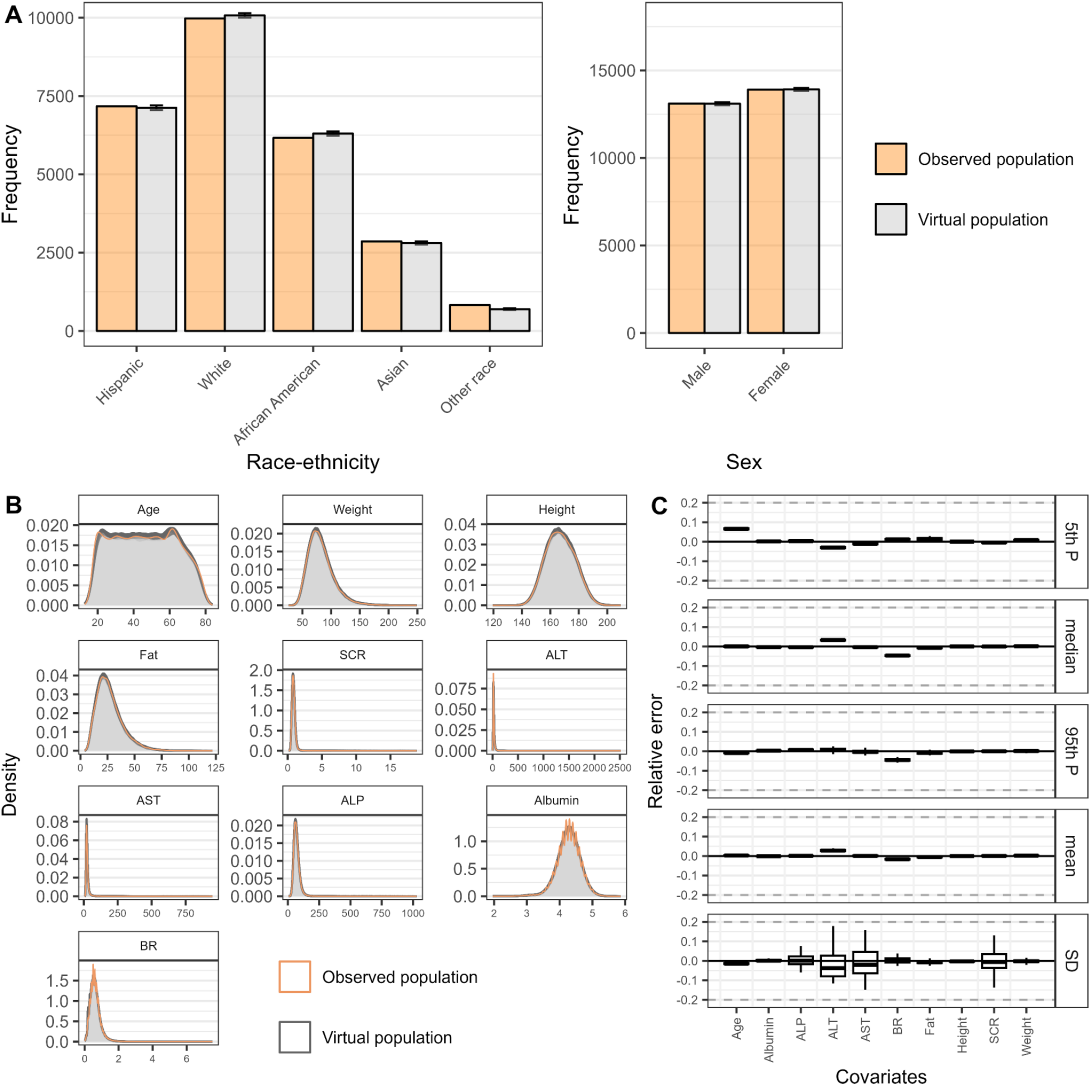
Marginal characteristics of the covariates in observed population and virtual populations simulated from NHANES copula. **A**. Frequency of each category in discrete covariates, race-ethnicity and sex, in the real-world population (orange columns) and virtual populations (grey columns). **B**. Density curves of each continuous covariate variable in the real-world population (orange line) and virtual populations (gray lines). **C**. Relative error of marginal metrics (percentiles, mean, and standard deviation) of continuous covariates as compared to the statistics of the real-world population. Virtual population was simulated 100 times. Error bars indicated the standard deviation of 100 simulations. Gray dashed lines indicate ± 20% relative error

The overall simulation performance of the developed copula model was evaluated for the entire population, without specifying any subgroups. For categorical covariates, (i.e., race-ethnicity and sex), the frequency of each category in the virtual population aligned with that of the real-world population (Fig. 2A). For continuous covariates, density curves of each individual covariate in the simulation dataset well tracked observed ones (Fig. 2B); mean, standard deviation and percentiles of VP agreed with those of the observed population, with relative errors within ± 0.10 (Fig. 2C). For percentiles and mean metrics, coefficient of variation across simulations were all within 0.007, and those of standard deviation were within 0.09.

**Fig. 3:**
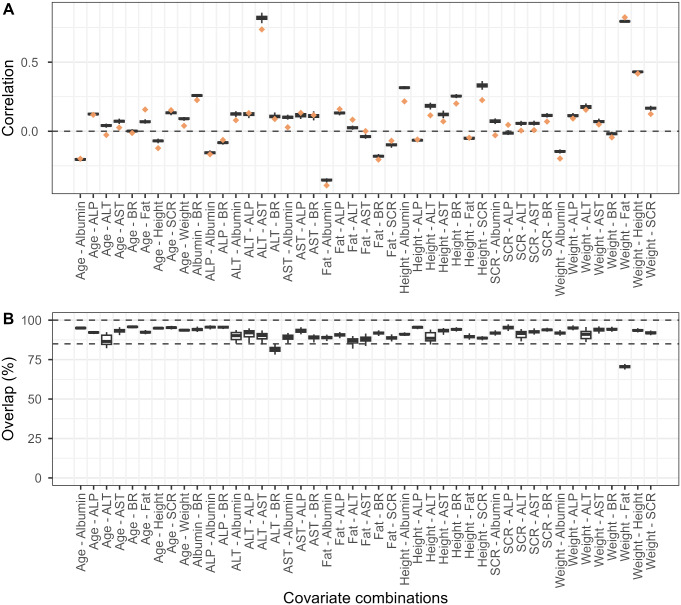
Dependency metrics of covariate pairs in the observed population and virtual populations simulated from NHANES copula. **A**. Correlations of each covariate pair in real-world population (orange diamond) and virtual populations (black box). Gray dashed line represents no correlation between covariate pairs. **B**. Overlap metric of 95th density contours of virtual population relative to observed population. Virtual population was simulated 100 times. Error bars indicated the standard deviation of 100 simulations. Gray dashed lines indicate 100% and 85% overlap percentages

The simulated correlations from the copula model were very similar to observed correlations for most pair combinations of covariates, with 0.023 median error (Fig. 3A). Covariate pairs associated with the largest error of correlation were height-SCR (0.105) and SCR-albumin (0.102). The median overlap was 92.0% across all covariate pairs and simulations, and the model achieved over 85% overlap in 96% (43/45) covariate pairs, indicating a good capture of dependency structure (Fig. 3B). The only two covariate pairs that did not reach 85% were ALT-BR and weight-fat, with 81.4% and 70.5% overlap percentages.

The full copula model reproduced the marginal properties as well as the dependence relations of covariates of input population data. Variability across simulations tended to be small for all metrics except for standard deviation, showing the robustness of the copula model.

### Subgroup performance

To gain further insights into the usefulness of the full copula for simulating subgroups of the total population, we conducted two separate investigations on the performance of full copula for VP simulation in race-ethnicity and sex subgroups.

#### Race-ethnicity subgroup analysis

The full copula was able to approximate the marginal characteristics of the observed population in Hispanic, White, and African American subgroups, with median relative errors of marginal metrics across covariates within [-0.19, 0.28] (Figure S3). For Asian and Other race VP populations, median relative errors were in the ranges [-0.21, 0.41] and [-0.68, 0.20], respectively. For comparison, subgroup copulas for Hispanic, White, African American and Asian populations showed good performances in terms of the marginal metrics, with the median relative errors of all covariates in [-0.10, 0.16]. However, the relative errors were larger for Other race subgroup copula, with a range of [-0.46, 0.09]. Full copula and subgroup copulas showed comparable performance in capturing the marginal attributes of Hispanic, White, African American and Other race subgroups, however, subgroup copula showed superior performance in Asian population.

**Fig. 4:**
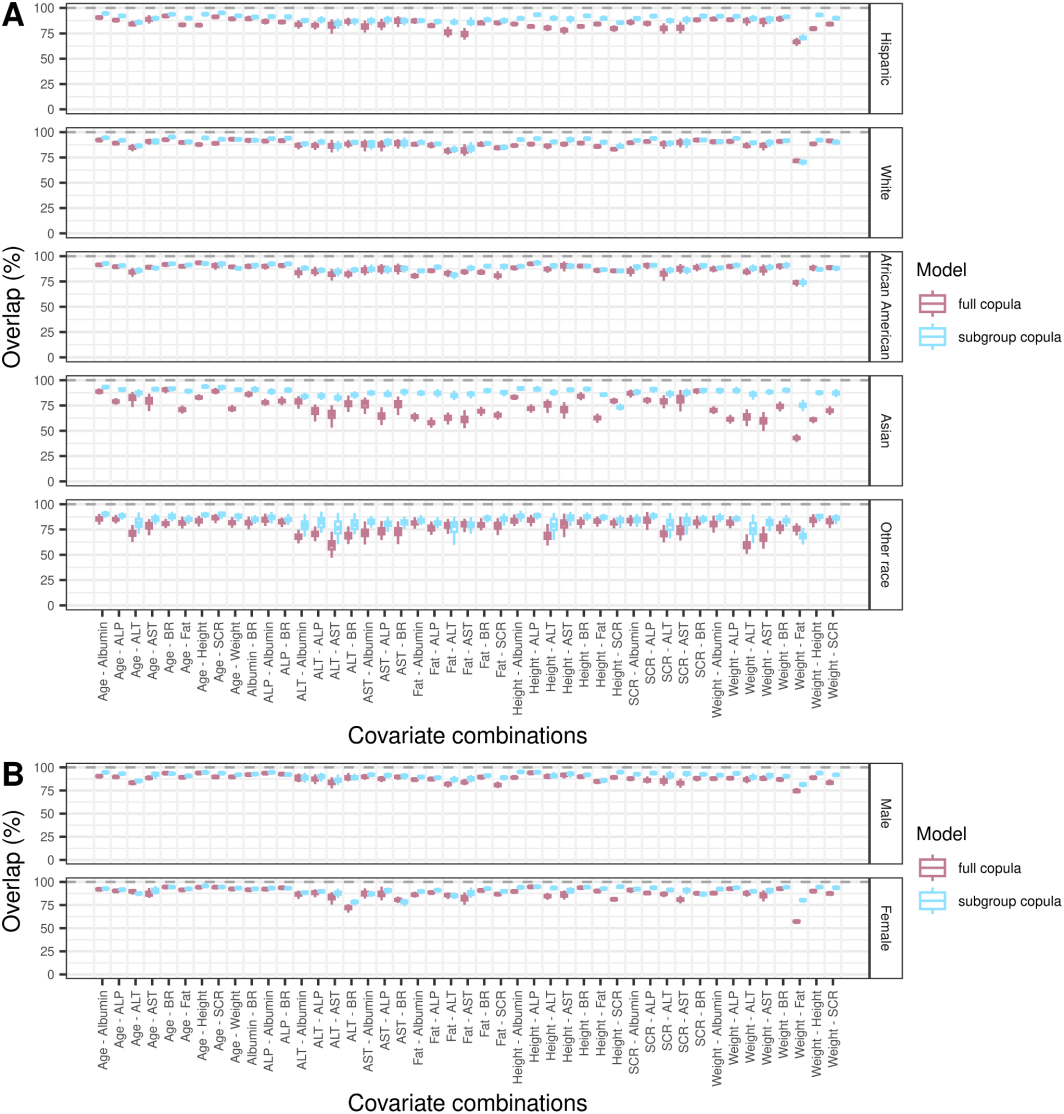
Overlap metric of each subgroup virtual population relative to corresponding real-world population. **A**. Overlap metrics calculated for each race-ethnicity subgroup population. **B**. Overlap metrics calculated for each sex subgroup population. The full copula was created utilizing the whole set of data, while subgroup copulas were developed based on each subgroup of data. Subgroup virtual populations were simulated 100 times using full copula (pink boxes) and subgroup copulas (blue boxes) for each. Error bars indicated the standard deviation of 100 simulations. Gray dashed lines indicate 100% overlap percentage

The full copula model achieved 84.6%, 88.6%, 87.8%, 74.0% and 80.1% median overlap percentages for Hispanic, White, African American, Asian and Other race populations, while subgroup copulas reached 89.7%, 91.0%, 89.0%, 88.7% and 85.1% (Fig. 4A), respectively. Subgroup copulas outperformed the full copula in simulating the dependence structure of covariates in Asian and Other race subgroups, but showed similar performance in the rest of race-ethnicity subgroups.

#### Sex subgroup analysis

In general, compared with subgroup copulas, the full copula model could well capture the margins and dependency structures in male and female populations. For marginal metrics, median relative errors of full copula were within the range [-0.19, 0.09] and [-0.28, 0.07] for male and female populations (Figure S4). For comparison, subgroup copulas for male and female yielded median relative errors of [-0.33, 0.06] and [-0.07, 0.08]. The median overlap metric of full copula was calculated to be 88.5% and 88.8% for male and female populations (Fig. 4B), while subgroup copulas achieved 91.9% and 92.1% overlap percentages for the two populations.

### R shiny application

**Fig. 5:**
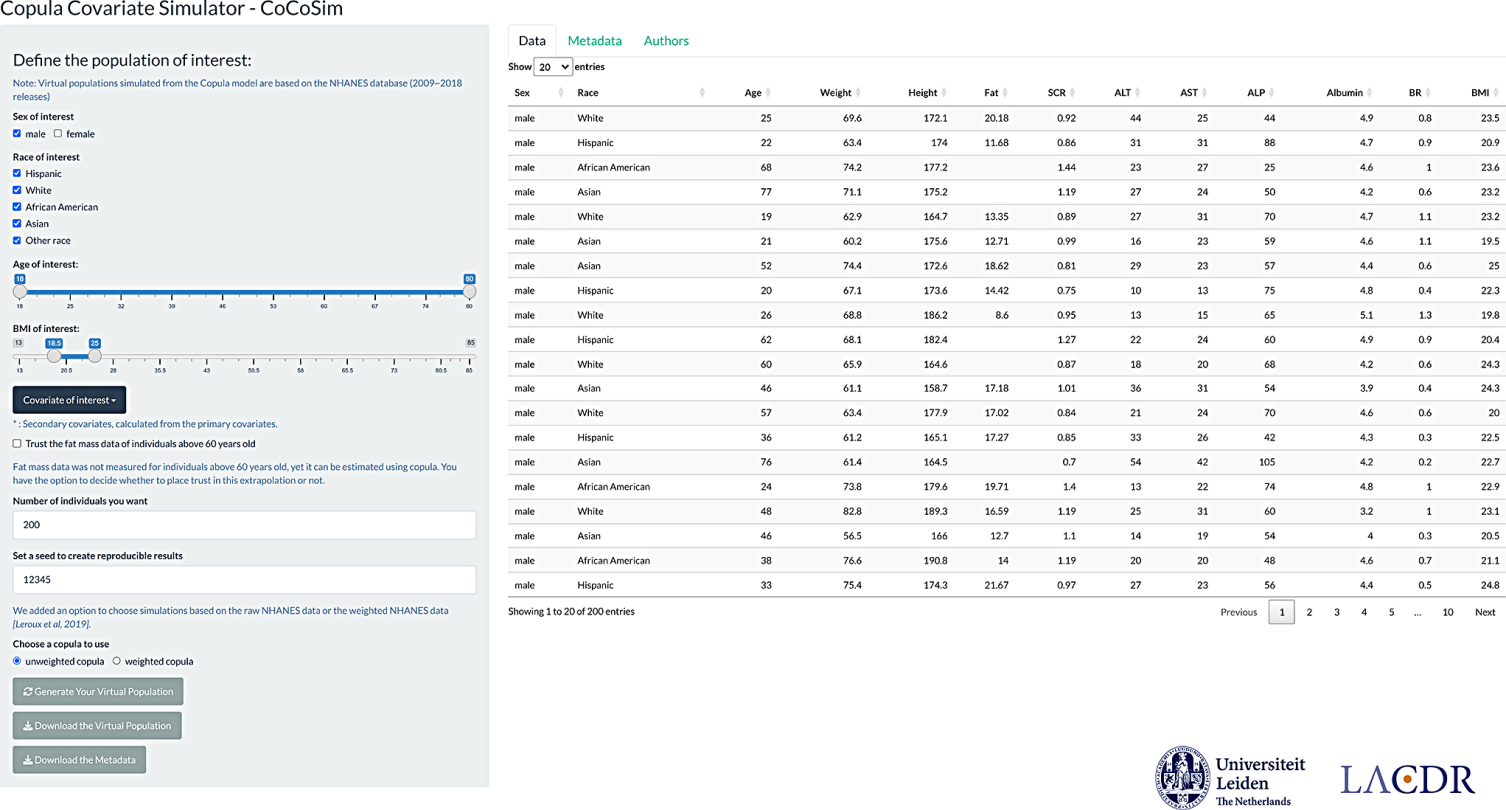
Interface of the R shiny application CoCoSim (https://cocosim.lacdr.leidenuniv.nl/). Virtual population can be generated according to user-defined characteristics based on NHANES copula (full copula)

The copula covariate simulator (CoCoSim) web application was developed based on the NHANES copula and made available online (https://cocosim.lacdr.leidenuniv.nl/, Fig. 5). Using this application, VPs can be generated online following these steps: (1) define the population of interest by selecting race-ethnicity, sex, age, and body mass index (BMI); (2) select the covariates of interest. Secondary covariates, including BMI, lean body weight, and estimated glomerular filtration rate, can be calculated based on the covariates in NHANES dataset; (3) select the number of individuals for simulation; (4) select the weighted or unweighted NHANES copula for the virtual population simulation; (5) generate the VP and download the data.

With the app, users can generate virtual population with desired characteristics, including race-ethnicity, sex, age and BMI ranges. Generated virtual populations can then be used as covariate distributions for pharmacometric model-based simulations, for example as part of clinical trial simulations or dosing strategy optimization simulations.

## Discussion

We developed a copula model for an adult population which adequately captured the covariate distributions as present in the NHANES database.

The tree structure of the NHANES copula revealed associations between commonly used covariates in population pharmacokinetics studies, which may help in the process of covariate model development. Identified associations were in line with the literature in which sex was found to influence height, weight, serum creatinine, and liver function biomarkers (total bilirubin and ALT) [30]. The correlation between covariates may explain the situations where sex may not be relevant as a covariate when the other covariates are included, since different PK or PD outcomes depend on underlying covariates (such as weight and serum creatinine) [31, 32].

To evaluate the performance of the developed copula model, we assessed whether the simulated population is realistic by comparing the marginal and dependency metrics between VPs and real-world populations. Interestingly, we observed that the pair combinations of covariates that showed the largest errors of correlation differed from those showing the lowest overlap percentages. Pearson correlation quantifies linear associations, while data sharing the same linear correlation could exhibit different dependency structures, and the overlap metric takes the shape or pattern of the dependency into account. Jaccard index is a similarity measure between two data samples [27], and the novelty of the overlap metric lies in its first application to two-dimensional densities. Pearson correlation and overlap metric collectively depicted the joint behavior at a pairwise level and addressed different perspectives, and as such should be evaluated together when assessing copulas or investigating the similarity between two populations.

The advantage of copulas is that they can model complex multivariate distributions more easily and efficiently. The local poor fit for dependency structure between fat-weight conditional on sex (Fig. 4B) is probably due to the parametric bivariate copulas used for the estimation of vine copulas. The fat-weight distribution of the real-world population exhibited a heart-shaped contour (Fig. 1B), which might be better captured by non-parametric bivariate copulas. Yet, deploying non-parametric copulas is computationally expensive and is prone to overfitting [33].

This study is focused on the adult. The pediatric population was not considered in this analysis mainly because some critical covariates for the pediatric population are lacking in NHANES database, such as birth weight, postnatal age and gestational age. Additionally, the pediatric population differs from the adult population in anatomical, physiological and biochemical characteristics [34], and the developmental changes over age may lead to drastic changes in the dependency structure between covariates. This could lead to inferior performance if the copula was estimated on both populations. We have thus chosen to focus on adults only.

In this study, we incorporated not only continuous but also categorical variables in the estimation of the NHANES copula. Currently, copula models for unordered categorical variables are not fully identifiable [24]. To include race-ethnicity (an unordered categorical variable) in copula, we estimated vine copulas by iterating through all possible orders of race-ethnicity and selected the model with the lowest AIC value. Since there were five categories in race-ethnicity, we considered 120 unique order possibilities of race-ethnicity categories, which was time-consuming and computationally expensive. Since this type of variable is common in clinical studies, such as disease classification, an algorithm that could efficiently deal with unordered categorical covariates is yet to be developed. When categorical variables are transformed to a uniform scale, each value does not have the same probability of occurring, and it is still inherently discrete. Instead of calculating correlations, we chose to perform a subgroup analysis for categorical variables.

Copula models can be useful to support model-based dosing optimization or clinical trial simulation. For such applications, a focus on subjects with specific covariate characteristics usually exists [35, 36]. To this end, it is important to confirm whether a copula model correctly reflects covariate distributions for relevant population subgroups of interest. In our analysis, compared with subgroup copulas, the full copula model showed comparable performance across different race-ethnicity and sex subgroups except for Asian and Other race subgroups, likely due to the relatively small number of individuals within the entire dataset. In particular, the Asian population has distinct marginal distributions of weight, height and logFat, compared to other race-ethnicity populations (Figure S5). This led to a stronger correlation between height and weight, the relation between which predominates the underlying dependency structure (Figure S6). The ability to adequately simulate subgroups from a large copula is of great importance since creating copulas for each subpopulation of interest, including e.g. different age and BMI ranges creates a nearly infinite amount of possible subgroups.

The NHANES population represents the non-institutionalized population of America and cannot be classified as healthy subjects or patients, indicating that the virtual population simulated from full copula should be interpreted with care. In this dataset, a significant portion of fat mass data was missing due to the age-eligible criterion (< 60 years old) of examination. However, copulas allowed for interpolation and extrapolation of VPs, as they support the generation of fat mass data for individuals above 60 years old via conditional density functions [37]. Of note, we removed the extrapolated fat mass data during the evaluation of copula performance. Although no significant bias was revealed in the validation analysis, simulated fat mass for people above 60 years old should be used with caution.

To make the full copula more accessible to the community, a web application was developed to facilitate the simulation of VPs with user-defined properties. The application allows to generate virtual populations with specific demographic attributes such as race-ethnicity, sex and BMI. Yet, the performance of NHANES copula on special populations, has not been able to be validated in this study. Additional data related to these populations are necessary to further investigate copulas for specific types of patients, such as pediatric, obese, pregnant, and renally impaired patients. This work served as a basis for building a copula library in future, for sharing the copulas of special patient populations and supporting simulation studies. Collaborative efforts could be initiated to gather large-scale data to build copulas for various target populations.

A copula can generate virtual populations that accurately represent the input population, and allows for adjusting sampling weights in the estimation in case the input population is not representative of the real-world population. In this way, copulas facilitate the generation of virtual populations that are representative of the actual real-world populations if sampling weights are available. The marginal distribution of unweighted and weighted copula differed mainly in different race-ethnicity groups (Figure S7). Our web application includes both unweighted and weighted copulas. In the analysis, we focused on the unweighted copula, because the comparison between the virtual population and the input population is only possible with the unweighted copula.

## Conclusion

In this study, we demonstrated the development and evaluation of a copula model using NHANES database to simulate commonly used covariates in pharmacometric modeling, which can be used as part of clinical trial design and dose strategies optimization. A user-friendly web application was developed to facilitate the use of the developed copula model for covariate simulation.

## Electronic supplementary material

Below is the link to the electronic supplementary material.

Supplementary Material 1

## Data Availability

No datasets were generated or analysed during the current study.
